# Augmented Reality Applications in Interventional Radiology: Possibilities and Challenges

**DOI:** 10.1007/s00270-023-03402-4

**Published:** 2023-03-22

**Authors:** Uli Fehrenbach

**Affiliations:** Department of Radiology, Charité Universitätsmedizin Berlin, Augustenburger Platz 1, 13353 Berlin, Germany

Technical innovations increasingly affect various areas of everyday life. A field that has come into focus in recent years, especially in lifestyle applications, is augmented reality (AR). AR claims to combine real-life with virtual content for an immersive experience known as “mixed reality”.

Besides entertainment, the AR approach is also being evaluated in various medical specialties to explore its potential as an assist device in patient treatment [1]. The classic use of AR in medicine applies to surgical disciplines [2]. Typical applications used by surgeons run on special high-end headsets that combine the real image of the surgical site with previously acquired radiological images and thus facilitate navigation. Initial limitations in implementation of the technology such as the bulky appearance of the devices and their cabling, as well as the insufficient computing power, are increasingly receding as further technical developments become available [3]. Nowadays, almost everyone carries a powerful minicomputer in the form of a personal smartphone in their pocket. This could enable the use of performance-intensive applications without the costly installation and purchase of hardware.

Besides surgery and medical education, AR tools have also been developed for interventional radiology [4]. The most extensively studied AR applications support imaging-guided punctures as presented in this manuscript by Morita et al. [5]. The smartphone AR application presented herein supports the interventionalist with a rotatable 3D live overlay of the puncture tract in out-of-plane CT-guided needle placements. Two different types of easy to perform image registration were investigated in a phantom study, and their results show that both methods achieved equally high accuracy with short procedure times. The use of the app in the technically demanding out-of-plane puncture technique, which can be challenging, especially for less experienced interventionalists, is worth highlighting. The main advantages of smartphone-based solutions over needle-guidance assist systems from the manufacturers of CT scanners would be their low initial cost and wide availability without the need for complex installations.

The main task for us as interventionalists is to scientifically evaluate the applicability and accuracy of those novel AR applications. And this is precisely where the greatest challenges lie. Anyone who performs CT-guided punctures knows that the main task of the interventionalist is to adjust for deviations from the originally planned puncture tract. This particularly affects organ systems that move in a respiratory-dependent manner, such as the liver and lungs. Preclinical studies on immobile phantoms cannot adequately represent this real-life scenario and simplify the technical challenges of image registration in patients. Thus, comparability usually exists only to immobile puncture targets, which also pose little challenge in routine clinical practice. Therefore, the main advantage of AR applications here would be to reduce radiation exposure, which would be straightforward and inexpensive with applications such as the one presented in this manuscript. On the other hand, the wider availability of the required hardware tends to give rise to “third-party” software solutions, which are usually only evaluated on a small scale. However, it is essential to evaluate the applications on a larger scale to provide recommendations and enable their translation into clinical routine. For this, we need extensive exchange within the IR community, which could be accomplished through our international societies, to enable further distribution and technical developments. Even though experienced interventionalists are critical of such applications, which in their view are not necessary, it is essential to investigate their potential. It is our task to transfer modern technologies into our clinical routine by evaluating them in a scientifically adequate way, and thus to further develop our field of expertise. Being open-minded to innovations moves our specialty forward and makes it even more attractive to future generations of interventionalists.
